# Evaluation of the Bioactive Compounds Found in Tomato Seed Oil and Tomato Peels Influenced by Industrial Heat Treatments

**DOI:** 10.3390/foods10010110

**Published:** 2021-01-07

**Authors:** Katalin Szabo, Francisc Vasile Dulf, Bernadette-Emőke Teleky, Panagiota Eleni, Christos Boukouvalas, Magdalini Krokida, Nikolas Kapsalis, Alexandru Vasile Rusu, Claudia Terezia Socol, Dan Cristian Vodnar

**Affiliations:** 1 Institute of Life Sciences, University of Agricultural Sciences and Veterinary Medicine Cluj-Napoca, Calea Manastur 3-5, 400372 Cluj-Napoca, Romania; katalin.szabo@usamvcluj.ro (K.S.); bernadette.teleky@usamvcluj.ro (B.-E.T.); 2 Faculty of Agriculture, Department of Environmental and Plant Protection, University of Agricultural Sciences and Veterinary Medicine Cluj-Napoca, Calea Manastur 3-5, 400372 Cluj-Napoca, Romania; 3 Laboratory of Process Analysis and Design, School of Chemical Engineering, National Technical University of Athens, Iroon Polytechneiou 9, Zografou Campus, 15780 Athens, Greece; peleni@central.ntua.gr (P.E.); bouk@chemeng.ntua.gr (C.B.); mkrok@chemeng.ntua.gr (M.K.); 4 Greek Canning Company S.A. “KYKNOS”, Tomato Factory, 72o klm Old National Road Patra-Pyrgos, 27200 Savalia Ilias, Greece; nikolas.kapsalis@gmail.com; 5 Biozoon Food Innovations GmbH, Nansenstrasse 8, 27572 Bremerhaven, Germany; rusu_alexandru@hotmail.com; 6 CENCIRA Agrofood Research and Innovation Centre, Ion Meșter 6, 400650 Cluj-Napoca, Romania; clausocol@yahoo.com; 7 Faculty of Food Science and Technology, University of Agricultural Sciences and Veterinary Medicine Cluj-Napoca, Calea Manastur 3-5, 400372 Cluj-Napoca, Romania

**Keywords:** antioxidant, β-carotene, heat treatment, industry, linoleic acid, lycopene, syringic acid, tomato seed oil

## Abstract

The circular economy action plan involves principles related to food waste reduction and integration of recovered nutrients to the market. In this context, the present study aims to highlight the valuable bioactive components found in tomato processing by-products (carotenoids, phenolic compounds and fatty acids) influenced by industrial pre-treatments, particularly cold break (CB) process at 65–75 °C and hot break (HB) process at 85–95 °C. The fatty acid profile of the tomato seed oil was examined by gas chromatography coupled to mass spectrometry (GC-MS), individual carotenoid and phenolic compositions were determined by high performance liquid chromatography (HPLC) and the viscoelastic properties were evaluated by rheological measurements. The physicochemical properties revealed appropriate characteristics of the tomato seed oil to fit the standards of generally accepted edible oils, for both CB and HB derived samples, however, significant qualitative and quantitative differences were detected in their phenolic composition and carotenoids content. Lycopene (37.43 ± 1.01 mg/100 mL) was a major carotenoid in the examined samples, linoleic acid was the main fatty acid (61.73%) detected in the tomato seed oil and syringic acid appeared to be one of two major phenolic acids detected in the samples of CB process. Our findings extend the boundaries of tomato processing industry by validating that tomato seed oil is a bioactive rich edible oil with additional health benefits, which can be integrated in functional food products.

## 1. Introduction

In 2017 the tomato-based products market (juice, paste and peeled tomatoes) was estimated around 8.7 billion dollars worldwide according to FAOSTAT (http://www.fao.org/faostat/en/#data, 2020), indicating that tomato is one of the most important agro-industrial crops. In 2018 the global tomato production overlapped 182 million tons and it is estimated that more than a quarter of the production underwent processing, rising approximately 5.4–9.0 million tons of by-products, however, clear statistics on this regard is quite difficult to collect [1].

The tomato processing industry generates three fractions of by-products, depending on the peeling method applied as well as on the form of the final product (tomato paste, crushed or peeled tomatoes, etc.). These fractions, namely tomato peels, tomato seeds and the mixture of these two together, called tomato pomace, sum up to 5–30% of the main product [2]. The final product cost includes the expenses related to the waste management supported by the manufacturers and implicitly increases the product price. Thus, the alternative solutions resolving the inconveniences caused by the wastes might be most appreciated by stakeholders and consumers and might, as well, reduce environmental discomfort caused by improperly discarded by-products, which favors microorganisms growth given the high moisture content [3].

In the context of a bio-based economy, which relies on the production of renewable biological resources and the conversion of these resources and waste streams into value-added products [4], the tomato processing by-products represent a low priced biomass with various integration possibilities [5]. The zero-waste action plan encourages the use of renewable energy in fields like biodiesel production [6], the cereal industry [7,8], coatings and packaging [9,10,11,12] including agro-industrial by-products. Numerous scientific studies underline the valuable bioactive compounds contained by the biomass remaining after industrialization of the tomatoes such as carotenoids, phenolic compounds, dietary fiber, polyunsaturated fatty acids and others [13,14]. Meanwhile, clinical trials confirm the positive effects on human health of certain molecules contained by tomato by-products such as lycopene, acting against reactive oxygen species and preventing some non-communicable diseases in human [15,16,17].

Carotenoids are natural pigments responsible for the yellow, orange and red color of many fruits and vegetables, with outstanding antioxidant properties. They are synthesized by plants and microorganisms but not by the human organism, consequently, the primary source of carotenoids for the human is related to dietary habits. An increased carotenoid intake enhances the immune system and reduces the risks of the onset of degenerative diseases for example, different cancer types, cardiovascular diseases, cataracts and macular degeneration [18]. Recent studies highlight that women with higher plasma concentrations of β–carotene and α–carotene are at lower breast cancer risk [19]. Furthermore, lycopene supplementation was found effective against aging-related inflammatory and oxidative stress-induced neurodegeneration [20,21]. Besides these effects, carotenoids have provitamin A activity and if incorporated as bioactive ingredients into food formulations can help to improve the final product’s shelf life and/or sensory properties [3,22,23].

Phenolic compounds are a large group of plant secondary metabolites which gained increased attention recently because of the growing body of evidence indicating the benefits of plant-derived phenolics on the prevention of a large variety of diseases [7,24,25]. They occur naturally in plants like grapes, green tea, olives, blueberries, nuts, cocoa and others, with the highest concentrations found in the fruit’s skins and many of them show strong antioxidant effect [26,27]. These bioactive molecules can be found in tomato by-products as well and they can preserve their quality during industrial treatments and, by their revalorization, can bring advantages in terms of food waste reduction, less environmental disturbance, more efficient processing of tomatoes and value-added products in food science.

Polyunsaturated fatty acids (PUFAs) are a group of fatty acids mainly localized in cell membranes with an important role in membrane fluidity and many physiological functions, like regulation of blood pressure or cell signaling. Humans can synthesize all fatty acids utilized by the body except for the two essential PUFAs, linoleic acid and alpha-linolenic acid [28]. Major dietary sources for these two essential PUFAs are plant oils, including tomato seed oil which contains linoleic acid as a major compound [29]. Previous research regarding the bioactive compounds found in the lipid fractions of agro-industrial wastes found that oils extracted from grape, guava, melon, passion fruit, soursop, pumpkin and tomato seeds contain tocopherols, phytosterols and phenolic compounds and have good antioxidant capacity [13]. Besides these biologically active constituents, the oils presented, predominantly, unsaturated fatty acids in their composition, being suitable for the food industry and, as well, for pharmaceutical and cosmetic fields.

To date, the recovery of bioactive molecules represents an underrated sector of agro-industrial processing, with challenges linked to efficient and green extraction methods and to effective pretreatments of the biomass to be extracted [30]. Earlier studies on bioactive compounds recovery from agro-industrial waste (apple peels, carrot pulp, white- and red-grape peels and red-beet peels and pulp) highlighted that thermal treatment enhanced the bioavailability of certain compounds and increased their total phenolic content [31]. Phytochemicals with antioxidant activities like tocopherols, polyphenols, carotenoids, some terpenes and sterols found in tomato processing by-products seem to resist industrial treatments, nominating tomato processing wastes as a source of natural bioactive molecules [32].

These results linked together served as a hypothetic base for our experiments conducted on the recovery and reutilization of industrial tomato by-products with the scope to identify in which direction thermal pre-treatment influences carotenoids and phenolics content of tomato peels and the fatty acid composition of the tomato seed oil. Earlier findings related to the bioactive compounds found in tomato waste examined the peels fraction [2] and tomato pomace fraction [29] and the results showed mean carotenoids content of 2.5 and 30.2 mg/100 g DW, respectively, however the provenience of the samples was not of industrial origin.

The aim of the present study is to evaluate carotenoids content of samples derived from the tomato processing industry influenced by two different thermal treatments, precisely hot break (HB) and cold break (CB) processing lines. Furthermore, the fatty acid profile and phenolic composition of the oil obtained from tomato seeds was investigated, in order to draw a clear picture about the possible revalorization of the by-products.

## 2. Materials and Methods

### 2.1. Chemicals and Standards

Ethyl acetate, petroleum ether, food grade hexane (ACS grade, ≥99%) used for extractions and methanol CHROMASOLV^®^ (gradient grade, for high performance liquid chromatography (HPLC), ≥99.9%), acetonitrile CHROMASOLV^®^ (gradient grade, for HPLC, ≥99.9%) for HPLC analysis were purchased from Sigma-Aldrich (Steinheim, Germany). Fatty acid and carotenoid standards, as well as chemicals used for oil extraction, fractionation and preparation of fatty acid methyl esters (FAMEs) and reagents implied in the experiments like Folin-Ciocâlteu regent, were of analytical grade, purchased from Sigma-Aldrich (Steinheim, Germany).

### 2.2. Sample Preparation

Tomato peels and seeds were collected from two production lines of KYKNOS canning company (Savalia Ilias, Greece), namely cold break (CB) and hot break (HB) processing lines. Samples from CB processing line, noted with A, stand for the tomato pomace obtained by preheating of cleaned and chopped tomatoes up to 65–70 °C temperature range, followed by juice extraction and separation of the by-product (process mainly used for concentrated tomato paste). Samples from HB processing line, noted with B, are the tomato pomace derived from preheated tomatoes up to 85–95 °C temperature range (process mainly used for ketchup or different types of tomato sauce and industrial use). The by-product (pomace mass—tomato seeds, peels and small amounts of pulp mixed together) collected from the two different production lines, was separated by sedimentation in water to obtain two different fractions, specifically tomato peels and seeds. Dried samples were received from industry in opaque sealed packages, to avoid carotenoids loss and further analyzed in the laboratories of Food Science and Technology at the University of Agricultural Sciences and Veterinary Medicine, Cluj-Napoca within two weeks from receiving time.

### 2.3. Analytical Measurements of the Tomato Peels

#### 2.3.1. UAE Procedure of Carotenoids from the Tomato Peels

Ultrasound assisted extraction (UAE) was applied to recover carotenoids from the tomato peels due to the advantages related to processing time and to the possibility of using low processing temperatures in recovery of heat-sensitive compounds like carotenoids. The samples (0.5 g tomato peels from both processing lines) were placed in Falcon tubes with 5 mL mixture of methanol/ethyl: acetate/petroleum: ether (1:1:1, *v*/*v*/*v*), underwent sonication in an ultrasonic unit (Elma Schmidbauer GmbH, Singen, Germany) with an ultrasonic power of 60 W/g at 37 kHz frequency for 10 min and were centrifuged for separation 5 min at 11,000 rpm [29]. The supernatant was collected in a separation funnel and the pellet was re-extracted two more times by the same procedure. The collected extracts were washed successively with sodium chloride solution (15%) and diethyl ether. The organic phase, containing the targeted carotenoids, was dried over anhydrous sodium sulphate and the solvent was evaporated by a rotary evaporator (Rotavapor R-124, Buchi, Flawil, Switzerland) at 35 °C and further analyzed by HPLC with a diode-array detector (DAD).

#### 2.3.2. Quantitative and Qualitative Analysis of Carotenoids by HPLC/DAD

The extracts were dissolved in 1 mL ethyl acetate, passed through a 0.45 µm pore size Millipore and injected into the HPLC/DAD system. Carotenoids were determined by an Agilent 1200 HPLC system coupled to diode array detector (Agilent Technologies, USA, CA, Santa Clara), using a reversed-phase EC 250/4.6 Nucleodur 300–5 C-18 ec. column (250 × 4.6 mm), 5 m (Macherey-Nagel, Germany) as previously described [2,29]. The mobile phases consisted of mixtures of acetonitrile:water (9:1, *v*/*v*) with 0.25% triethylamine (A) and ethyl acetate with 0.25% triethylamine (B). The gradient started with 90% A at 0 min to 50% A at 10 min; the percentage of A decreased from 50% at 10 min to 10% A at 20 min. The flow rate was 1 mL/min and the chromatograms were registered at 450 nm. Individual carotenoids were quantified by using the calibration curve of a β-carotene standard from Sigma-Aldrich (Steinheim, Germany).

### 2.4. Analytical Measurements of the Tomato Seeds

#### 2.4.1. Simple Solvent Extraction of Oil from Tomato Seeds

Prior to the extraction of tomato seed oil, the fraction of dried seeds of the two processing lines was powdered and sieved (800–1000 μm). The tomato seed oil was obtained using simple solvent extraction with food grade hexane 1:10 *w*/*v* under continuous stirring (300 rpm) for 2 h at room temperature. The obtained extract was taken to a rotary evaporator under vacuum (100 mbar) at a temperature of 38 °C to remove the hexane and collect the oil. The oil samples were further characterized by physical, chemical and rheological measurements.

#### 2.4.2. Physical Chemical Characterization of the Tomato Seed Oil

Tomato seed oils were characterized by the assessment of refractive index (RI), determined according to AOAC method (AOAC 921.08, 2000), iodine value (IV), relative density and peroxide value (PV), these parameters are generally used as quality indicators of edible oils [33]. Briefly, iodine value measures the amount of unsaturation content or double bonds which can react with iodine and it was expressed as grams of iodine consumed by 100 g of tomato seed oil. Relative density (RD) or specific gravity is a dimensionless quantity of a substance expressed as

RD = ρ_substance_/ρ_reference,_ (1)

where ρ_substance_ is the density of tomato seed oil and ρ_reference_ is the density of water at room temperature. Peroxide value can be used as an oxidative index for the early stage of lipid oxidation and it is expressed in units of milliequivalents (meq) of free iodine per kilogram of oil.

#### 2.4.3. Total Carotenoids Content of Tomato Seed Oil

The analysis of the total carotenoids concentration was performed by UV-Vis spectrophotometry according to the methodology described by da Silva & Jorge (2014) [13], briefly an aliquot of tomato seed oil was dissolved in hexane 1:1 (*v*/*v*) and underwent UV-Vis spectrophotometry (Jasco V-530). The absorption was measured at 450 nm and the quantification of total carotenoids was calculated by absorption in the wavelength of 450 nm, specific for β-carotene and with an absorptivity value of 2592, in hexane. The values were expressed as mg of β-carotene per 100 mL of oil (mg β-carotene/100 mL).

#### 2.4.4. Quantification of Individual Carotenoids Found in the Tomato Seed Oil by HPLC/DAD

An aliquot of tomato seed oil was dissolved in hexane (1:1 *v*/*v*), passed through a Millipore filter (0.45 μm pore size) and injected to the HPLC/DAD system. Individual carotenoids were determined as described earlier in Section 2.3.2.

#### 2.4.5. Total Phenolic Content of Tomato Seed Oil

Total phenolic content (TPC) of the oil samples was determined by the Folin-Ciocâlteu assay as previously described [34]. This method estimates the total content of all phenolic compounds and measures the reducing capacity of a sample. In order to test their reducing power, aliquots of 25 µL methanolic extracts were mixed with 1.8 mL distilled water in a 24 well microplate. An aliquot of 120 µL of Folin-Ciocâlteu reagent was added to the wells and mixed, followed by the addition of 340 µL sodium carbonate solution (7.5% *w*/*v*), to create basic conditions between phenolic compounds and Folin-Ciocâlteu reagent for the redox reaction. After incubation in the dark for 60 min at room temperature, the absorbance was read at 750 nm using a microplate reader (BioTek Instruments, Winooski, VT, USA). The analysis was repeated three times and results were expressed as mg gallic acid equivalent (GAE)/100 mL tomato seed oil.

#### 2.4.6. Quantitative and Qualitative Analysis of Phenolic Compounds Found in Tomato Seed oil by HPLC-DAD-ESI-MS

The phenolic composition of the samples was determined by HPLC with diode array detection and electrospray ionization mass spectrometry (HPLC-DAD-ESI-MS) using an Agilent 1200 HPLC system (Agilent Technologies, Santa Clara, CA, USA). The system was equipped with an Eclipse column, XDB C18 (4.6 × 150 mm, 5 mm) and the binary gradient, consisted of 0.1% acetic acid:acetonitrile (99:1) in distilled water (*v*/*v*) for solvent A and 0.1% acetic acid in acetonitrile (*v*/*v*) for solvent B, was eluted at a flow rate of 0.5 mL/min, following an elution program described previously [34]. The phenolic compounds were monitored by DAD and the absorption spectra (200–600 nm) were collected continuously during each run. The working conditions for MS fragmentation were ESI (+) module with a scanning range between 100 and 1200 *m*/*z* full-scan option. Capillary voltage was set on 3000 V, at 350 °C with a nitrogen flow of 8 L/min. Data analysis was performed using Agilent ChemStation Software (Rev B.04.02 SP1, Palo Alto, CA, USA) and the total phenolic content was calculated as the sum of individual concentrations of the phenolic components.

#### 2.4.7. Assessment of Total Lipid Content of the Tomato Seeds

Folch’s extraction procedure was used to determine the total lipid content of tomato seeds with slight modifications. As previously described [29], a sample of tomato seeds (3 g) from each processing line (CB and HB) was homogenized in 5 mL of methanol for 1 min using a high-power homogenizer (MICCRA D-9, ART Prozess-und Labortechnik, Mullheim, Germany); then, 10 mL of chloroform was added and the homogenization continued for 2 more min. The mixture was filtered and the solid residue was re-extracted with chloroform/methanol (2:1, *v*/*v*, 15 mL). The filtrates obtained were combined and washed with 0.88% aqueous potassium chloride, in a separation funnel. The purified lipid (bottom) layer was filtered and dried over anhydrous sodium sulphate and the solvent was removed in a rotary evaporator. Total lipid content was determined gravimetrically.

#### 2.4.8. Fatty Acid Profile of the Tomato Seed Oil

An aliquot (10–15 mg) of the tomato seed oils was transesterified into FAMEs, using an acid-catalyzed procedure, as described previously [34] and analyzed by GC-MS. A PerkinElmer Clarus 600 T GC–MS (PerkinElmer, Inc., Shelton, CT, USA) system was used, equipped with a Supelcowax 10 capillary column (60 m × 0.25 mm i.d., 0.25 μm film thickness; Supelco Inc., Bellefonte, PA, USA). The column temperature was programmed from 140 to 220 °C at a rate of 7 °C/min and held for 23 min. The carrier gas (helium) was adjusted to a constant flow rate of 0.8 mL/min and the mass spectra were recorded in EI (positive ion electron impact) mode. The mass scans were performed from 22 to 395 *m*/*z*. Identification of the fatty acids was made by comparing peak areas with known standards and compounds listed in an MS database using NIST MS Search 2.0 software.

The quantitative analysis of FAMEs was performed using the total ion current chromatograms and the prevalence (%) of each fatty acid was computed as follows:

FA% = IFA/Ʃ TAFA × 100,(2)

where IFA is individual fatty acid area and TAFA is total area of fatty acids.

#### 2.4.9. Rheological Measurements of the Tomato Seed Oil

The effect of two types of heat treatment (CB and HB) on tomato seed derived oil was investigated with an Anton Paar MCR 72 rheometer (Anton Paar, Graz, Austria). The rheometer was equipped with a Peltier plate-plate system (P-PTD 200/Air) with temperature control and a 50 mm diameter smooth parallel plate geometry (PP-50-67300) [35]. The measurement of the viscosity curves was effectuated at different shear rates between 5–300 s^−1^, with logarithmic stepwise presets. Samples (~3 mL tomato seed oil) were poured straight on the lower plate and preset at different temperatures (4, 10, 20, 30, 40, 50, 60, 70, 80, 90, 95 °C). The gap between the plates was set at 1 mm and the sample surplus was removed before measurements.

### 2.5. Statistical Analysis

The analytical measurements were carried out in triplicate (*n* = 3) and the results are presented as mean values ± standard deviation (SD). The collected data sets were subjected to analysis of variance (ANOVA) and further examined by Tukey’s multiple comparison test at a confidence level of *p* < 0.05 to detect significant differences between the means. Data processing was carried out using computer program Excel 2016 (Microsoft Office^®^).

## 3. Results and Discussion

### 3.1. Carotenoids Content of Tomato Peels Derived from Two Production Lines (CB and HB)

**Figure 1:**
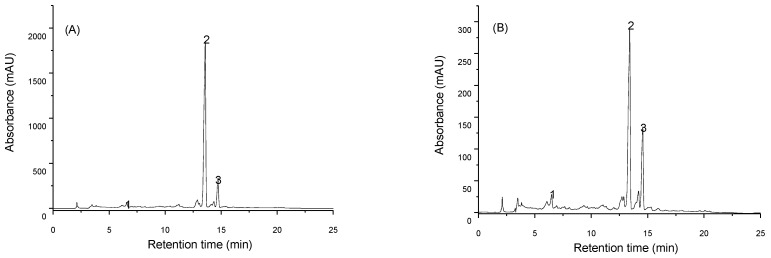
Identification of individual carotenoids recovered from tomato peels of cold break (**A**) and hot break (**B**) processing lines. 1—lutein, 2—lycopene, 3—β-carotene.

In the present study UAE was applied to recover carotenoids from tomato peels originated from two different industrial processing lines, namely CB and HB processing. Individual carotenoids of the samples were identified by HPLC-DAD and two of the most representative chromatograms are shown in Figure 1.

The quantitative analysis of lycopene, lutein and β-carotene was made by mass spectrometry and the results are given in Table 1. Samples exposed to CB process presented a mean lycopene content of 41.04 ± 1.24 mg/100 g DW while those exposed to HB process had 9.53 ± 0.62 mg/100 g DW indicating a statistically significant (*p* < 0.05) degradation of lycopene during industrial pre-treatment at 85–95 °C. Previous studies related to tomato waste analysis reported lycopene content ranging between 13.40 and 81.54 mg/100 g [36], however, according to Strati and Oreopoulou [16], a much wider variation exists in reported extraction yields of lycopene, due to factors like tomato variety, tomato processing method, by-product fraction, extraction techniques and others.

We investigated total and individual carotenoids content of tomato peels, knowing that major part of these pigments are found in the exocarp of tomatoes, which is removed during industrial processing and disposed. According to Knoblich, Anderson and Latshaw [34] the lycopene level in tomato peel is 73.4 mg/100 g while in tomato seed is 13.0 mg/100 g, as well, Al-Wandawi and colleagues found that lycopene concentration is approximately three times higher in tomato peels than in whole ripe tomatoes [37] acknowledging tomato peels as a valuable source of natural carotenoids. Furthermore, other studies suggest that removal of tomato peels and seeds during home cooking or processing results in a loss of their potential health benefits [38].

Degradation kinetic of lycopene is more accentuated than β-carotene’s, as the results show a mean β-carotene content of 5.03 ± 0.33 mg/100 g DW in the samples provided from CB process and 3.41 ± 0.12 mg/100 g DW for the ones from HB process. The lutein content of the samples from preheated tomatoes up to 85–95 °C exhibited a statistically significant decrease (0.65 ± 0.05 mg/100 g DW) compared to those from the CB processing line (1.54 ± 0.04 mg/100 g DW) when the tomatoes were preheated up to 65–70 °C.

The carotenoids content of the samples from CB process was significantly higher (47.61 ± 1.61 mg/100 g DW) than that recorded in the samples obtained from the HB process as shown in Table 1 (13.59 ± 0.73 mg/100 g DW). The thermal degradation of lycopene and β-carotene in tomatoes during hot air drying was previously investigated and the results showed an accelerated degradation by increasing the temperature from 70 °C to 80 °C [39]. Our findings follow the same tendency with respect to lycopene degradation ascertaining that HB process significantly affects the individual and total carotenoids content in tomato peels.

The major bioactive components found in tomato are carotenoids, which have well documented health-promoting functions in the human organism [15,40,41]. Lycopene, especially, has been shown as an efficient singlet oxygen quencher with approximately twice the activity of β-carotene, having good cardio-protective effects [42]. Carotenoids can serve as natural pigments in the food industry, lycopene and β-carotene are authorized for this purpose and can be utilized as natural antioxidants for functional foods or as additives in different food matrices to elongate their shelf-life [18], therefore tomato peels derived from industrial processing can constitute an inexpensive source of natural colorants with additional health promoting properties.

### 3.2. Characterization of Tomato Seed Oil Derived from Two Production Lines (CB and HB)

#### 3.2.1. Physical Chemical Characteristics

Total lipids content of the tomato seeds was determined by simple solvent extraction and the mean yield was 19.1 ± 0.2 percent *w*/*w* on a dry basis. The content of tomato seeds in oil ranges between 20 and 37% *w*/*w* on a dry basis and is differentiated by the variety of tomato and the pretreatment applied [43,44]. Several physicochemical characteristics of the oil samples such as relative density, iodine value, refractive index are presented in Table 2 together with the Codex Alimentarius CXS 210–1999 standard values for vegetable oils [33].

These parameters are generally used as quality indicators for edible oils and both CB and HB process derived samples fall into the range of the Codex standard. The total phenolic content of the samples was evaluated, as per Folin-Ciocâlteu method and expressed as mg GAE/100 mL oil. The CB process derived samples presented significantly higher values (333 ± 0.2 mg GAE/100 mL oil) compared to HB process derived samples (102 ± 0.1 mg GAE/100 mL oil), indicating a strong effect of heat treatment on the total phenolic content of the samples. Phenolic compounds are free radical scavengers playing important roles in oxidation processes and previous research related to the bioactive compounds of the lipid fractions show a rich phenolic composition of agro-industrial by-products, helping to protect the oils against lipid oxidation [13].

The total carotenoids content of the oil samples was determined by UV-Vis spectrophotometry (Supplementary Material Figure S1) and a statistically significant (*p* < 0.05) difference was observed between the samples, more exactly the CB process derived samples had 16.77 ± 2.09 mg β-carotene/100 mL oil and the HB process derived samples had 8.14 ± 1.48 mg β-carotene/100 mL oil. Further investigations were made by HPLC-DAD for a more accurate identification of individual carotenoids in the samples.

#### 3.2.2. Identification of Individual Carotenoids by HPLC/DAD

Individual carotenoids were determined from the oil samples and are presented in Table 3. Lycopene was predominantly present in the samples with a mean value of 37.43 ± 1.01 mg/100 mL oil from the CB process and of 22.01 ± 0.74 mg/100 mL oil from the HB process, emphasizing a statistically significant difference between the heat treatments. β-carotene was identified and quantified in amounts of 9.35 ± 0.62 mg/100 mL and 6.63 ± 0.38 mg/100 mL oil from the CB and HB process, respectively, showing a significant decrease in the samples derived from HB process. Lutein was found, as well, in 1.16 ± 0.01 mg β-carotene/100 mL oil and 1.10 ± 0.04 mg β-carotene/100 mL oil, amounts in the CB and HB processing line derived samples, respectively and it seems that heat treatments does not significantly affect the lutein content of the samples.

Carotenoids play a significant role in human health through their complex biological activities, however, it is known that factors like dietary habits, “species of carotenoids” and/or host-related factors can strongly modulate carotenoids bioavailability [45]. To ensure a higher absorption rate in the human organism, carotenoids need to be escorted by lipids because they can facilitate the extraction of carotenoids from the food matrix and can stimulate micelle production through biliary secretion, a necessary feature for enhanced absorption rate of the carotenoids. The results of the present study show a high carotenoids content of the tomato seed oil—47.94 ± 0.40 mg/100 mL oil from the CB process and 29.73 ± 0.76 mg/100 mL oil from the HB process—therefore, it can constitute a good source of daily intake of carotenoids with the additional advantage of a higher absorption rate due to their dissolution in lipid fraction.

The difference between the total carotenoids measured by UV-Vis and HPLC could be explained by the compounds identified by each of the two methods. According to the methodology [13], the first method, UV-Vis, aimed to identify the total compounds absorbing at wavelength 450 nm specific to β-carotene; however, as it can be observed in the supplementary material 1, lycopene had the maximum absorption rate, at wavelength 470 nm [46], therefore strongly influencing the total carotenoids content and resulting in the differences when compared to HPLC analysis. This analyze adds important value to the samples full characterization. By the HPLC methodology [29], based on higher accuracy, we have identified each individual carotenoid within the samples, such as β-carotene, lycopene and lutein, resulting in the observed variation in results.

Thermal treatment of the tomatoes influences the quantity of carotenoids found in the seed oil and shows a significant decrease in lycopene and β-carotene but not in lutein content. This fact could be attributed to a higher thermal resistance of lutein and to its hydroxyl group, as well, which is more polar than β-carotene and may possibly less react in a nonpolar solvent like hexane [47]. Lutein is considered to have anti-inflammatory properties and a diet supplemented with lutein could prevent the occurrence of some ocular disease [48]. Previous research on the thermal and storage characteristics of tomato seed oil suggests that special attention needs to be turned to light exposure and storage temperature of the tomato seed oil given their sensitivity to these parameters [49]. The same study presents high thermal stability and excellent physicochemical profile for tomato seed oil, thus indicating a good potential to fully develop and produce tomato seed oil in industry as a desirable edible oil.

The sustainable valorization of tomato processing by-products remains a subject of great concern in the context of bio-economy and circular economy and considerable research efforts are focused on this topic [1], however further achievements need to be made for acceptance by consumers. Integration of tomato processing by-products was previously reviewed and numerous products like bakery, pasta and noodles, dairy, meat and oil products as well as jams and even functional ice cream were supplemented with different fractions of the tomato industrial by-products [5]. An important aspect to consider in the creation of new food formulation is the general acceptance of consumers and, therefore, careful ingredient selection, dosage and formulation is essential.

#### 3.2.3. Rheological Measurements

**Figure 2:**
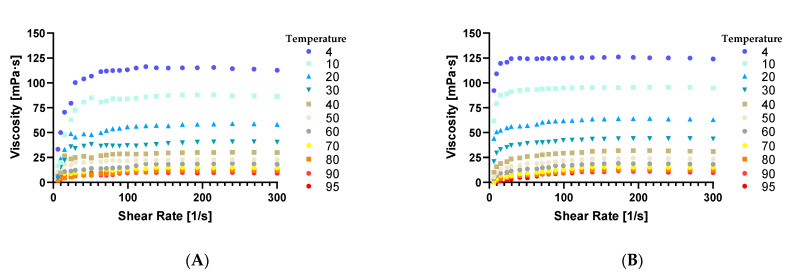
The viscosity of extracted tomato seed oil derived from (**A**) cold break method (**B**) hot break (**B**) method between 4–95 °C (Graph Prism Version 8.0.1).

Rheological property variations of oil extracted from tomato seed with two distinct methods were evaluated at shear rates between 5–300 s^−1^, which present the relation of viscosity and shear rate (Figure 2A,B).

In food production oils play an important role in the textural and rheological characteristics of the final product [50]. Internal frictional forces in fluids can be measured by viscosity and consequently, the flow resistance as well. The viscosity of oils found in commerce act as ideal Newtonian liquids in which the viscosity decreases at a rapid pace with the increase of the temperature [51]. Shear rate (preset at values between 5–300 s^−1^) is established by the flow velocity of the sample split by the shear gap diameter (in our case 1 mm). Figure 2 presents the relationship between viscosity and the shear rate of the extracted tomato seed oil at various temperatures. Through the increase of temperature, the viscosity of the samples decreased in both cases, which indicate that both samples are Newtonian fluids.

In comparison, the oil obtained from the CB process had a lower viscosity than the oil obtained from the HB process, which is in accordance with the existing literature [52,53]. Nevertheless, in both cases, the viscosity decreased with the increase of temperature especially from 4 °C to 40 °C, from 113 mPa·s decreased to 30 mPa·s. At temperatures higher that 50 °C the viscosity ranged between 10–22 mPa·s which was also observed in similar studies that analyzed polysaccharides in black tomato pomace [54]. Several types of oils have the same decreasing trend in viscosity (under 25 mPa·s) with the increase of temperature [55,56]. The decrease in viscosity can be explained by the fact that higher temperatures promote the motion of molecules and diminishes the forces between them, consequently is enabled the movement of each layer between each other [57].

The viscosity of extracted tomato seed oil in both cases was higher than in the case of chia seed oil, with values for the CB of 52.2 mPa·s and HB of 63.5 mPa·s at 20 °C. The viscosity of chia seed oil was 47.5 mPa·s at the same temperature. The viscosity of tomato seed oil decreased substantially reaching values of 29.5 mPa·s for CB processing derived samples (Figure 2A) and 31.9 mPa·s for the samples derived from HB processing (Figure 2B) at 40 °C while chia seed oil viscosity decreased to values of 41.5 mPa·s [58]. The same or higher viscosity (31.6–52.6 mPa·s) was observed in the case of several edible oils, like sesame, palm, soybean, olive [51] and purslane seed oil [59].

In tomato juice production HB and CB changes in viscosity are given by the pectin structure of the obtained product. Through HB process a product with greater viscosity can be obtained, which could be explained through the inactivation of the pectolytic enzyme. In comparison over the CB method the pectins are just partially deteriorated, resulting a product with a lower viscosity [53]. In the extracted oil the viscosity can be described through their fatty acid profiles. Several studies report increased viscosity with the increase of saturated fatty acids and a decrease with the increase of unsaturated fatty acids [60,61]. With HB method the percentage of PUFAs was higher and the percentage of MUFAs was lower, than with the CB method, which led to a higher viscosity in our case.

#### 3.2.4. Fatty Acid Profile of the Tomato Seed Oil

The fatty acid composition of the oil samples, investigated by GC-MS, are presented in Table 4. The major components were linoleic acid (C 18:2 *n*-6), oleic acid (C 18:1 *n*-9) and palmitic acid (C 16:0). Smaller amounts of stearic (C 18:0) (<4%) and very small or trace percentages of hexadecenoic (C 16:1 *n*-9), vaccenic (C 18:1 *n*-7), α-linoleic (C 18:3 *n*-3) and arachidic (C 20:0) acids were also identified in the samples derived from both processing lines.

The major fatty acid identified was linoleic acid, representing 61.73% in CB process derived samples and 64.63% in HB process derived samples, with no statistically significant differences (*p* < 0.05) between the two types of heat treatments. However, a possible mechanism involved in the slight increase of linoleic acid in HB derived samples might be attributed to a higher degradation of the cell wall of tomato seeds at temperature of 85–95 °C, therefore a higher release of the linoleic acid from the matrix. Moreover, there is a slight possibility that thermal treatment of the tomatoes (85–95 °C) inactivated the enzymes responsible for the linoleic acid degradation [62] and explains a higher PUFAs content in the HB derived samples.

Linoleic acid is a polyunsaturated *n*-6 fatty acid, fundamental for human nutrition and it is an important component of animal and plant cell membranes, therefore, the main source for its intake is through diet. The findings of a study conducted on male Golden Syrian hamsters examined plasma and hepatic cholesterol-lowering effects of tomato processing by-product and the results suggests that tomato seed oil may be a good source of polyunsaturated fat for human consumption without the risk of hypercholesterolemia and obesity [63].

The quality and digestibility of vegetal origin edible oils are driven by the quantity and composition in unsaturated fatty acids [13]. Earlier findings related to the fatty acid profile of the lipid fractions extracted from agro-industrial wastes reported UFA/SFA ratio values between 3 and 7.3 for grape, guava, melon, passion fruit, pumpkin, tomato and soursop wastes, tomato seed oil rating 4.4. Our findings show 4.4 UFA/SFA proportion for the oil samples from CB processing line and an increased value, 5.2 for the HB processing line. These results places tomato seed oil in between pumpkin (3.0) and passion fruit (5.9) seed oils concerning UFA/SFA ratio.

#### 3.2.5. Qualitative and Quantitative Analysis of the Phenolic Composition by HPLC-DAD-ESI-MS

The phenolic composition of the samples was determined by HPLC-DAD-ESI-MS and the individual phenolic compounds are presented in Table 5. Five components from the phenolic class were identified in the CB process derived oil samples, namely caffeic acid-glucoside isomer (CG), caffeic acid (CA), syringic acid (SyA), di-caffeoylquinic acid (di-CQA) and tri-Caffeoylquinic acid (tri-CQA). The major components were di-CQA, a conjugated chlorogenic acid with several isomeric forms having great antioxidant properties and SyA, known for its biological activities like, antimicrobial, anti-inflammatory, neuro and hepatoprotective activity. Di-CQA can be found in roasted coffee beans, along with other chlorogenic acids that are linked to reduced risks of developing different chronic diseases due to their non-enzymatic antioxidant activities. The mechanism of action of these compounds relies on donating hydrogen atoms to reduce free radicals and to inhibit oxidation reactions [64].

The sum of individual phenolic compounds identified was 4.194 mg chlorogenic acid equivalent/L oil in the CB process derived samples and it decreased significantly to 1.950 mg chlorogenic acid equivalent/L oil in the HB process derived samples. The HB process seems to affect the tomato seed oils composition, quantitatively and qualitatively, as CG and CA were not detected in the samples derived from HB process. According to the existing literature, CA and CG are sensitive molecules to higher temperatures [65] and their heat sensitive nature made them decrease to a non-detectable level in HB samples. Previous studies regarding tomato seed oil for edible use and the effects of CB and HB processes over the physicochemical properties found that temperature did not significantly influence the oil samples [43], however, it has to be mentioned that, individual phenolic composition or other sensitive measurements like HPLC were not examined in the study. Interestingly, the CB process is recommended by authors for industry, based on economic considerations. Our findings indicate that CB process provides higher quality tomato seed oil than HB process, emphasizing a more complex phenolic composition of the samples derived from the processing line with mild temperature treatment.

## 4. Conclusions

Our study examined the by-products derived from two industrial processing lines of tomatoes, precisely cold break and hot break heat treatments. The tomato peels suffered significant modifications in their carotenoids content because of the hot break process, as revealed by approximately 76 percent lycopene degradation. The tomato seed oils were investigated by their physicochemical characteristics and the obtained results met the standards indicated by the Codex Alimentarius for vegetable oils [33], for both cold break and hot break derived samples. However, a consistent difference was observed in the total phenolic content of the samples assessed by Folin-Ciocâlteu method and further investigations by high performance liquid chromatography disclosed significant qualitative and quantitative variation in their phenolic composition. Significant changes were detected in carotenoids content of the oil samples as well, influenced by the thermal treatments. The fatty acid composition showed a slight increase of the poly-unsaturated fatty acids in the hot break derived samples. By-products of the tomato processing industry are a rich source of carotenoids, phenolic compounds and fatty acids which have relevant health promoting functions in the human organism. These bioactive compounds can be significantly impacted by the thermal treatment applied during industrialization, nominating cold break process more suitable for their preservation. Further research needs to be focused on the tomato seed oils acceptance by consumers and health benefits.

## Figures and Tables

**Table 1:** Individual and sum of carotenoids identified in tomato peels derived from cold break and hot break processing lines, determined by high performance liquid chromatography (HPLC) with a diode array detector and expressed as mg/100 g DW; analytical measurements were conducted with three replicates (*n* = 3).

Compound	λmax (nm)	Rt (min)	Samples of	
			Cold Break	Hot Break
			Content (mg/100 g DW)	Content (mg/100 g DW)
Lutein	448, 474	6.41	1.54 ± 0.04 *	0.65 ± 0.05
Lycopene	446, 473	13.42	41.04 ± 1.24 *	9.53 ± 0.62
β Carotene	455, 480	14.51	5.03 ± 0.33 *	3.41 ± 0.12
Sum of carotenoids			47.61 ± 1.61 *	13.59 ± 0.73

Means ± standard deviation followed by * indicate statistically significant differences between homogenous groups in line according to Tukey’s test (*p* < 0.05).

**Table 2:** Physical-chemical properties of tomato seed oils derived from cold break and hot break production lines; analytical measurements were conducted with three replicates (*n* = 3).

	Refractive Index	Relative Density	Iodine Value (g I/100 g oil)	Peroxide Value (meq/kg oil)	Total Carotenoids (mg/100 mL)	Total Phenolic Content (mg GAE/100 mL)
Sample						
Cold break	1.4723	0.890	44	<5	16.77 ± 2.09 *	333 ± 0.2 *
Hot break	1.4720	0.925	35	<5	8.14 ± 1.48	102 ± 0.2
Codex standard 210	1.448–1.477	0.890–0930	4–150	< 5		

Means ± standard deviation followed by * indicate statistically significant differences between homogenous groups in column according to Tukey’s test (*p* < 0.05).

**Table 3:** Individual and sum of carotenoids identified in tomato seed oil derived from cold break and hot break processing lines, expressed as mg β-carotene/100 mL oil; analytical measurements were conducted with three replicates (*n* = 3).

Compound	λmax (nm)	Rt (min)	Samples of	
			Cold Break	Hot Break
			Content (mg/100 g DW)	Content (mg/100 g DW)
Lutein	448, 474	6.41	1.16 ± 0.01	1.10 ± 0.04
Lycopene	446, 473	13.42	37.43 ± 1.01 *	22.01 ± 0.74
β Carotene	455, 480	14.51	9.35 ± 0.62 *	6.63 ± 0.38
Sum of carotenoids			47.94 ± 0.40 *	29.73 ± 0.76

Means ± standard deviation followed by * indicate statistically significant differences between homogenous groups in line according to Tukey’s test (*p* < 0.05).

**Table 4:** Fatty acid composition (%) of tomato seed oil derived from cold break and hot break production lines; analytical measurements were conducted with three replicates (*n* = 3).

Fatty Acids	Rt (min)	Sample	
		Cold Break	Hot Break
C 16:0	22.154	14.42	12.43
C 16:1n-9	22.430	0.07	0.09
C 18:0	25.391	3.95	3.59
C 18:1n-9	25.991	17.88	17.33
C 18:1n-7	26.031	0.31	0.37
C 18:2n-6	27.091	61.73	64.63
C 18:3n-3	28.352	1.5	1.39
C 20:0	29.752	0.13	0.17
	Ʃ SFAs	18.51	16.19
	Ʃ MUFA	18.26	17.79
	Ʃ PUFA	63.23	66.02
	Ʃ *n*-3 PUFA	1.5	1.39
	Ʃ *n*-6 PUFA	61.73	64.63
	UFA/SFA	4.41	5.2
	PUFAs/SFAs	3.42	4.08

C16:0—palmitic, C16:1 *n*-9—7-hexadecenoic, C18:0—stearic, C18:1 *n*-9—oleic, C18:1 *n*-7—vaccenic, C18:2 *n*-6—linoleic, C18:3 *n*-3—α-linolenic, C20:0—arachidic, SFAs—saturated fatty acids, UFA—unsaturated fatty acids, MUFAs—monounsaturated fatty acids, PUFAs—polyunsaturated fatty acids.

**Table 5:** Individual and sum of phenolic compounds of tomato seeds oils derived from cold break and hot break processing lines, expressed as mg chlorogenic acid equivalent/L oil; analytical measurements were conducted with three replicates (*n* = 3).

Compound	λmax (nm)	Rt (min)	Samples of	
			Cold Break	Hot Break
			Content (mg/100 g DW)	Content (mg/100 g DW)
Caffeic acid-glucoside isomer (CG)	292, 245	11.12	0.741 ± 0.01	n.d.
Caffeic acid (CA)	324, 250	12.54	0.555 ± 0.02	n.d.
Syringic acid (SyA)	290	15.05	1.122 ± 0.02 *	0.547 ± 0.02
Di-Caffeoylquinic acid (di-CQA)	328, 250	16.98	1.113 ± 0.01 *	0.812 ± 0.03
Tri-Caffeoylquinic acid (tri-CQA)	328, 250	20.31	0.662 ± 0.01	0.591 ± 0.04
Sum of phenolic compounds			4.194 *	1.950

Means ± standard deviation followed by * indicate statistically significant differences between homogenous groups in line according to Tukey’s test (*p* < 0.05); n.d.—not determined.

## Data Availability

Data is contained within the article or supplementary material.

## Supplementary Materials

The following are available online at https://www.mdpi.com/2304-8158/10/1/110/s1, Figure S1: UV-Vis spectra of carotenoids found in tomato seed oil derived from two production lines, cold break (CB) and hot break (HB).
