# Supplementary material for: Evaluation of the Bioactive Compounds Found in Tomato Seed Oil and Tomato Peels Influenced by Industrial Heat Treatments
Source: Foods. 2021 Jan 7;10(1):110. doi: 10.3390/foods10010110 (PMC7825722; doi:10.3390/foods10010110)
Supplement: Supplementary file 1 [file foods-10-00110-s001.pdf]

Figure S1

UV-Vis spectra of carotenoids found in tomato seed oil derived from two production lines, cold break (CB) and hot break (HB).

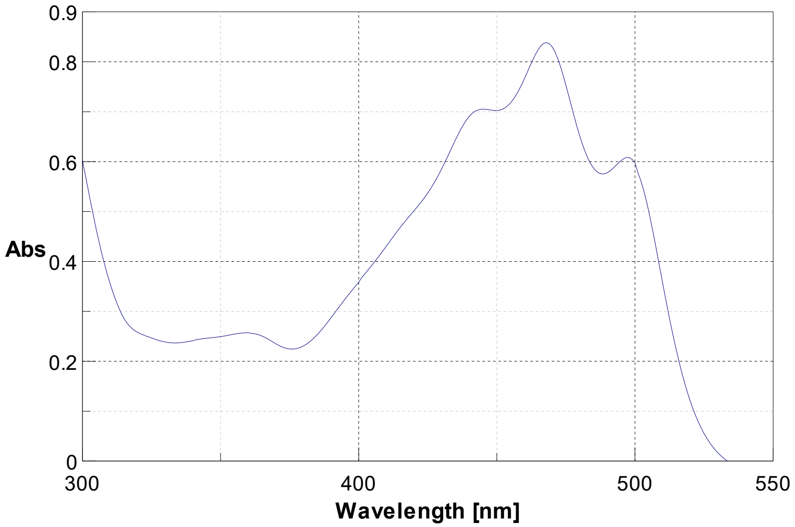

UV-Vis spectra of sample derived from CB production line

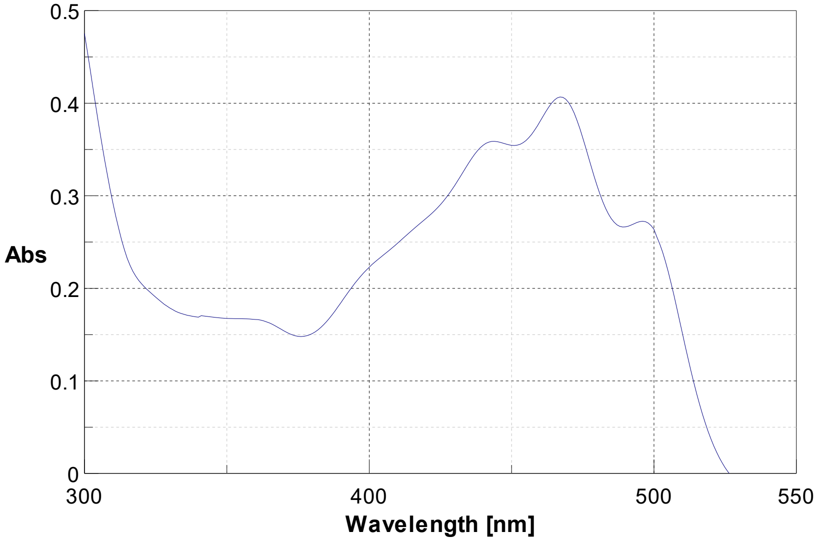

UV-Vis spectra of sample derived from HB production line
